# Mouse DC-SIGN/CD209a as Target for Antigen Delivery and Adaptive Immunity

**DOI:** 10.3389/fimmu.2018.00990

**Published:** 2018-05-07

**Authors:** Sjoerd T. T. Schetters, Laura J. W. Kruijssen, Matheus H. W. Crommentuijn, Hakan Kalay, Jordi Ochando, Joke M. M. den Haan, Juan J. Garcia-Vallejo, Yvette van Kooyk

**Affiliations:** ^1^Department of Molecular Cell Biology and Immunology, VU University Medical Center, Amsterdam, Netherlands; ^2^Department of Medicine, Icahn School of Medicine at Mount Sinai, New York, NY, United States; ^3^Immunología de Trasplantes, Centro Nacional de Microbiología, Instituto de Salud Carlos III, Madrid, Spain

**Keywords:** CD209a, dendritic cell-specific intercellular adhesion molecule-3 grabbing non-integrin, SIGNR5, vaccination, dendritic cell, antigen delivery

## Abstract

The efficacy of vaccination studies aimed at targeting antigens to human DC-SIGN (hDC-SIGN) have been notoriously difficult to study *in vivo*, as eight dendritic cell-specific intercellular adhesion molecule-3 grabbing non-integrin (DC-SIGN) homologs have been described in mice. CD209a/SIGNR5 has been coined as the mouse DC-SIGN (mDC-SIGN) ortholog, based on its expression and location in the genome. Nonetheless, which properties of hDC-SIGN are covered by mDC-SIGN is poorly investigated. One of the most important functions of DC-SIGN is the induction of adaptive immunity. As such, the aim of this study is to determine the capability of mDC-SIGN to induce adaptive immune responses. Here, we show that mDC-SIGN is expressed on GM-CSF cultured bone marrow-derived dendritic cells (BMDCs) and macrophages. However, mDC-SIGN is an internalizing receptor which, unlike hDC-SIGN, quickly resurfaces after internalization. Binding of OVA-coupled anti-mDC-SIGN antibody by BMDCs leads to quick internalization, processing, and presentation to antigen-specific CD8^+^ and CD4^+^ T cells, which can be boosted using the TLR4 ligand, monophosphoryl lipid A. In the homeostatic condition, mDC-SIGN is mostly expressed on myeloid cells in the skin and spleen. A subcutaneous injection of fluorescent anti-mDC-SIGN reveals specific targeting to mDC-SIGN^+^ skin dendritic cells (DCs) and monocyte-derived DCs *in situ*. A subcutaneous vaccination strategy containing OVA-coupled anti-mDC-SIGN antibody generated antigen-specific polyfunctional CD8^+^ T cell and CD4^+^ T cell responses and a strong isotype-switched OVA-specific antibody response *in vivo*. We conclude that mDC-SIGN shows partly overlapping similarities to hDC-SIGN and that targeting mDC-SIGN provides a valuable approach to investigate the immunological function of DC-SIGN *in vivo*.

## Introduction

The human innate immune receptor dendritic cell-specific intercellular adhesion molecule-3 grabbing non-integrin (DC-SIGN) recognizes pathogen- and host-derived glycoproteins (1). In addition, it mediates antigen internalization, processing, and presentation of antigens to T cells, which are functional hallmarks of antigen-presenting cells (APCs). Therefore, targeting antigens to human DC-SIGN (hDC-SIGN) has been shown to induce adaptive immune responses *in vitro* (2). In humans, DC-SIGN can be found on immature dendritic cells (DCs) and macrophages in peripheral tissues like the placenta and lung (3), as well as mature DCs in lymphoid tissue (4), but not on other APC subsets, including plasmacytoid DCs or Langerhans cells (5). DC-SIGN can also be found on DCs and M2-like macrophages in tumor tissue (6, 7) and on inflammatory macrophages in rheumatoid arthritis synovium (8). Interestingly, DC-SIGN expression is particularly high on monocyte-derived dendritic cells (moDCs) and dependent on IL4 (9). Although the physiological relevance of moDCs in humans is still unclear, in mice these cells have shown to contribute to antigen presentation and T cell activation (10). Although eight DC-SIGN-related receptors are described in mice, the absence of a clear murine ortholog has hampered the *in vivo* validation of hDC-SIGN and has so far been performed with mice that express hDC-SIGN driven by the CD11c promoter (11). Subsequent targeting of antigens in this model has demonstrated the potency of hDC-SIGN on CD11c^+^ DCs to internalize, process, and present antigen to T cells (12, 13). For example, targeting of DC-SIGN in combination with genetic depletion of regulatory T cells was sufficient to induce long-term tumor regression in B16 melanoma-bearing mice (14). A similar strategy induced high levels of antigen-specific CD8^+^ and CD4^+^ T cells, which protected mice from *Listeria monocytogenes* (15). While it is evident that hDC-SIGN is an effective gateway to strong adaptive immunity, its expression on all CD11c^+^ cells limits its translational value as an *in vivo* model for antigen targeting.

Of the eight mouse homologs, SIGNR5/CD209a has been coined as mouse DC-SIGN (mDC-SIGN) because of similar expression patterns and localization in the genome (16). Several reports have shown mDC-SIGN to be mostly expressed by moDCs, which are present in steady-state muscle (17) and skin (18) or develop from circulating monocytes after pro-inflammatory signals like GM-CSF (19), LPS (20), or even T cell activation (21). While mDC-SIGN^+^ moDCs have been shown to be potent inducers of adaptive T cell immunity, it still remains unclear whether mDC-SIGN itself is able to mediate antigen uptake and presentation to T cells.

Here, we show data that support the paradigm that mDC-SIGN shares expression patterns *in vitro* and *in vivo* with hDC-SIGN, as well as functional properties, including endocytic capacity and antigen presentation to CD8^+^ and CD4^+^ T cells *in vitro*. Combining targeting of antigen to mDC-SIGN and a potent adjuvant *in vivo* generates antigen-specific CD8^+^ and CD4^+^ T cells and increased antibody responses. In particular, targeting antigen to mDC-SIGN induces significantly higher antigen-specific humoral responses.

## Materials and Methods

### Mice

Mice transgenic for hDC-SIGN, OT-I, and OT-II on the C57BL/6 background have been described previously (11, 22, 23). The transgenic and wild-type C57BL/6 mice were bred at the animal facility of VU University (Amsterdam, Netherlands) under specific pathogen-free conditions and used at 8–16 weeks of age. Female and male mice were equally divided among groups, unless stated otherwise. All experiments were approved by the Animal Experiments Committee of the VU University and performed in accordance with national and international guidelines and regulations.

### Flow Cytometry Facilities and Reagents

All flow cytometry experiments were performed at the O_2_ Flow Facility at VU University (Amsterdam, Netherlands) using an X20 Fortessa flow cytometer (BD Biosciences) and ImageStreamX (Amnis Corp.) imaging flow cytometer. All antibodies were purchased from Biolegend, Miltenyi, and eBioscience (ThermoFisher), specifically: anti-CD4 (Clone GK1.5), anti-CD8 (Clone H35-17.2), anti-CD11b (Clone M1/70), anti-B220 (Clone RA3-6B2), anti-Ly6C (Clone HK1.4), anti-CD11c (Clone N418), anti-NK1.1 (Clone PK136), anti-CD45 (Clone 30-F11), anti-CD3 (Clone 145-2C11), anti-CCR2 (Clone SA203G11), anti-GR1 (Clone RB6-8C5), anti-CCR7 (clone 4B12), anti-mDC-SIGN (Clone MMD3), anti-MHCII (Clone M5/114.15.2), anti-CD16/32 (Clone 93), and Fixable viability dye-eFluor 780 (Thermo Fisher). OVA_257–264_-H2-Kb-PE tetramers were a kind gift from Dr. J. W. Drijfhout at the LUMC, Leiden, Netherlands.

### Imaging Flow Cytometry and Sample Preparation

Bone marrow-derived dendritic cells (BMDCs) were cultured as described by Lutz et al. (24). Because of the high number of cells needed for image flow cytometry, no *ex vivo* isolated DCs could be used in these experiments. BMDCs were incubated with anti-mDC-SIGN:AF488 antibody (clone MMD3) for 1 h, either on 4°C or 37°C. Cells were washed with PBS twice and fixed for 15 min using cold 4% PFA. After washing twice, the fixed cells were resuspended in PBS. Cells were analyzed on the ImageStream X100 (Amnis-Merck Millipore) imaging flow cytometer as previously described (25). A minimum of 15,000 cells were acquired per sample. The internalization score was calculated as previously described (25). Briefly, cells were acquired on the basis of their area. Analysis was performed with single cells after compensation (with a minimum of 5,000 cells). For standard acquisition, the 488-nm laser line was set at 100 mW. First, a mask was designed based on the surface of cells in the bright field image. This mask was then eroded to exclude the cell membrane. Finally, the resulting mask was applied to the fluorescence channel. The internalization score was then calculated on this mask using the Internalization feature provided in the Ideas v6.0 software (Amnis-Merck Millipore). Internalization can be interpreted as a log-scaled ratio of the intensity of the intracellular space vs. the intensity of the entire cell. Cells that have internalized antigen typically have positive scores, while cells that show the antigen still on the membrane have negative scores. Cells with scores around 0 have similar amounts of antigen on the membrane and in intracellular compartments.

### Mouse Tissue Collection, Digestion, and FACS Staining

Mice were sacrificed and skin-draining lymph nodes (LNs), spleen, skin, and blood were obtained for further analysis. Skin-draining LNs were verified by the presence of migratory DCs after 100 μl adjuvant [25 μg agonistic CD40 (in house 1C10) in 1:1 AddaVax (InVivoGen)] injection subcutaneously in the skin. For antigen-tracking experiments, skin biopsies were taken using 8-mm sterile dermal biopsy punches (KAI Medical) 2 h after injection of fluorescently labeled antibody with adjuvant. LNs, spleen, and skin were cut small using sterile scissors in 385 µg/ml liberase TL (2WU) and incubated at 37°C for 20 min. Enzymes were deactivated using ice-cold RPMI 1640 complete (10% FCS, 1% 50 U/ml penicillin, 50 µg/ml streptomycin, HEPES/EDTA). After digestion, cells were run through a 100-µm cell strainer and extensively washed before FACS staining. Cells were stained for 30 min at 4°C using only directly labeled primary antibodies and in the presence of 1 µg/ml anti-CD16/32 antibody. After extensive wash with PBS, labeled cells were fixed with 1% PFA at 4°C for 15 min, washed, and measured on the flow cytometer.

### Flow Cytometry Analyses

Flow cytometry data were analyzed first using FlowJo analysis software. First, files were compensated using UltraComp eBeads (Thermo Fisher) microspheres labeled with the appropriate antibodies. Compensation was additionally verified using fluorescence-minus-one (FMO) controls for every single fluorochrome for every tissue type (equally pooled per group) on experimental samples. Next, first gating was performed on a stable flow (time vs. cell count), subsequently on viability dye-negative/CD45-positive cells and finally on single cells (FSC-A/FSC-H). The resulting cells were concatenated and exported per experimental group into an FCS. file and uploaded to the Cytobank online analysis platform (https://www.cytobank.org/). Using the ViSNE module, we generate tSNE plots per tissue type based on the following input and analysis settings: all cells (concatenated) per condition used up to 300,000 total, number of iterations = 3,000, Perplexity = 50, Theta = 0.5. Cells were clustered by MHCII, CD11b, CD11c, B220, NK1.1, Ly6C, GR-1, and CD3 expression. Next, we identified and manually gated subpopulations as represented by the tSNE clustering analysis (Figure S2 in Supplementary Material), color-coded, and overlaid the subpopulations as represented in the graphs (Figure 4A; Figure S2 in Supplementary Material). After defining gating strategies, the individual experimental samples were similarly gated in FlowJo and statistics were exported to GraphPad Prism 6 for visualization. Histograms were generated in FlowJo by comparison of “fluorescence minus one” (FMO; all antibodies minus one) or isotype antibody as negative control.

### Antigen Presentation Assays

Bone marrow-derived dendritic cells were cultured as described by Lutz et al. (24). OTI and OTII transgenic mice were sacrificed, and spleens were mechanistically run through a 100-µm cell strainer. Red blood cells were lysed using ACK lysis buffer (0.15 M NH_4_Cl, 10 mM KHCO_3_, 0.1 mM EDTA) and washed before purification using MagniSort Mouse CD4- or CD8-negative isolation kits according to the manufacturer’s instructions (eBioscience/Thermo Fisher). Purified CD4^+^ and CD8^+^ T cells were labeled using 2μM CFSE and counted before co-culture. BMDCs and purified T cells were co-cultured for 3 days at 37°C, stained, and measured on an X-20 Fortessa flow cytometer. To avoid overestimation of CFSE-based proliferation results (26), we adopt the % responding cells metric. Results are calculated and represented as percentage responding cells (“calculated cells at the start of culture”/“number of cells that went into division” × 100). Total # of cells at start of culture = #G0 + (#G1/2) + (#G2/4) + (#G3/8)+ (#G4/16) + (#G5/32) + (#G6/64). Number of cells that went into division = “Total # of cells at start of culture” − G0.

### Generation of Ovalbumin-Coupled Antibody

In this study, we have used two clones of mDC-SIGN-binding antibodies (MMD2 and MMD3) with identical properties to visualize the receptor and investigate functional characteristics of the mDC-SIGN molecule (27). Anti-DC-SIGN (clone AZN-D1, clone MMD2) and IgG2c (clone 6.3; SouthernBiotech) antibodies were conjugated to ovalbumin (OVA; Calbiochem, Darmstadt, Germany) using the cross-linking agent sulfosuccinimidyl-4-(*N*-maleimidomethyl)-cyclohexane-1-carboxylate or Alexa Fluor 488 NHS Ester (20,000; Thermo Fisher) according to the manufacturer’s protocol (Pierce). Antibody conjugates were separated from reaction-reductants using PD-10 desalting columns (Pierce, Rockford, IL, USA). The concentration of OVA and antibody was determined using the bicinchoninic acid assay (Pierce) and ELISA. The products were tested for endotoxins using the Limulus Amebocyte Lysate assay and a level of <0.125 EU/ml was deemed acceptable.

### Vaccination

Mice were subcutaneously injected with endotoxin-free 25 μg anti-mDC-SIGN:OVA or anti-mDC-SIGN:AF488 with 25 μg agonistic CD40 antibody (in house, clone 1C10) in 1:1 PBS/AddaVax emulsion according to the manufacturer’s instructions (InvivoGen) in a maximum volume of 100 µl. For functional readouts, spleens and blood were collected 7 days after vaccination. For antigen-tracking experiments, organs were harvested 2 and 12 h after vaccine injection. For mDC-SIGN phenotyping experiments, C57BL/6 mice were sacrificed and organs harvested as described.

### OVA-Specific Antibody Determination

To determine the antigen-specific antibody response to the ovalbumin, NUNC Maxisorp 96-well plates (Thermo Fisher) were coated with 10 μg/ml purified ovalbumin (Sigma-Aldrich) for 24 h at 4°C in coating buffer in PBS. Next, plates were washed extensively with PBS/Tween 0.05% and additionally blocked with 1% PBS/BSA. A dilution range of serum, obtained from vaccinated mice (on day 7 after vaccination) through a heart puncture, was incubated over night at 4°C. After washing, samples were incubated with anti-mouse IgG-biotin (and anti-IgG1, 2, 3 isotypes) antibodies for 1 h at RT and after wash incubated with HRP-conjugated streptavidin for 1 h at RT. Then after washing, the ELISA plate was developed using TMB substrate buffer. Reaction was stopped when properly developed using 2N H_2_SO_4_ and extinction was measured at 450 nm using an iMark microplate reader (Bio-Rad). Serum dilution of 1:400 showed the most consistent and reproducible signal to noise ratio. All samples were normalized with PBS as blanco. Secondary antibodies (1:2,000) used: IgG1 115-065-205 (Jackson ImmunoResearch), IgG2a m32215 (Invitrogen), IgG2b ab97248 (Abcam), IgG3 1100-08 (ITK), IgM 62-6840 (Zymed), IgG 315-065-006 (Dianova), and Streptavidin-HRP p0397 (Dako).

### Statistics

Statistics were performed using GraphPad Prism 6 software. For the comparison of two groups, a Student’s *t*-test was used. For more than two groups, a two-way analysis of variance was used followed by a Tukey *post hoc* analysis to compare means between two groups. *P < 0.05, **P < 0.01, ***P < 0.001, ****P < 0.0001, data represented as mean ± SEM.

## Results

**Figure 1:**
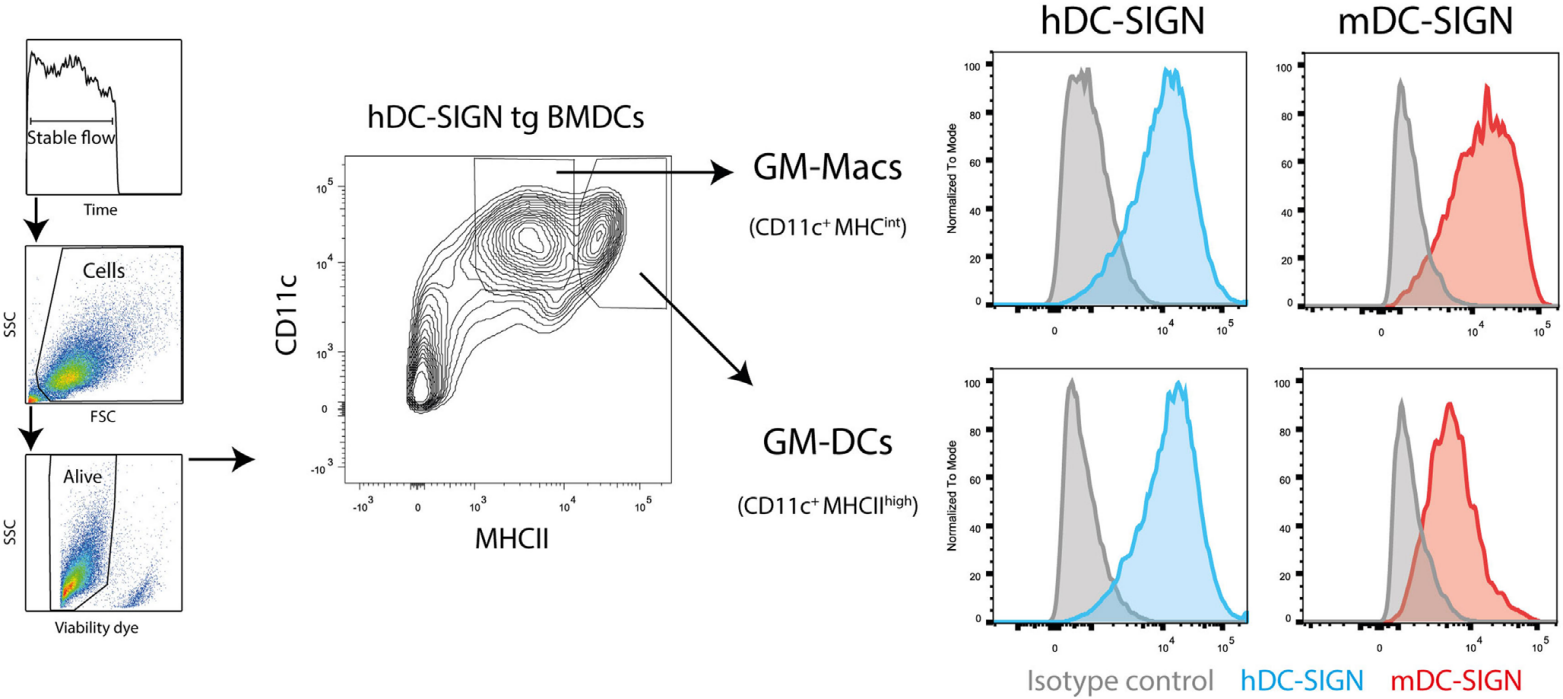
Mouse dendritic cell-specific intercellular adhesion molecule-3 grabbing non-integrin is expressed by GM-DCs and GM-Macs cultured *in vitro* cultures. Both GM-CSF-cultured (differentiation for 7 days) bone marrow-derived macrophages (GM-Macs) and dendritic cells (GM-DCs) express mDC-SIGN. Data are representative of two individual experiments.

First, to study the functional characteristics of mDC-SIGN, we examined its expression on cultured DCs *in vitro* by flow cytometry. We generated BMDCs, as previously described (24), using bone marrow from hDC-SIGN transgenic mice (11). GM-CSF-cultured BMDCs roughly generate two populations of APCs distinguished by CD11c and MHC class II expression, GM-DCs (CD11c^+^MHCII^high^), and GM-Macs (CD11c^+^MHCII^int^) (28). Both classically GM-CSF-cultured BMDCs and macrophages showed clear expression of mDC-SIGN and hDC-SIGN (Figure 1). No difference in mDC-SIGN expression between WT and hDC-SIGN transgenic was observed, while hDC-SIGN was absent on wild-type BMDCs, as expected (data not shown). Interestingly, mDC-SIGN is higher expressed on GM-Macs compared with GM-DCs cultured from bone marrow precursors.

**Figure 2:**
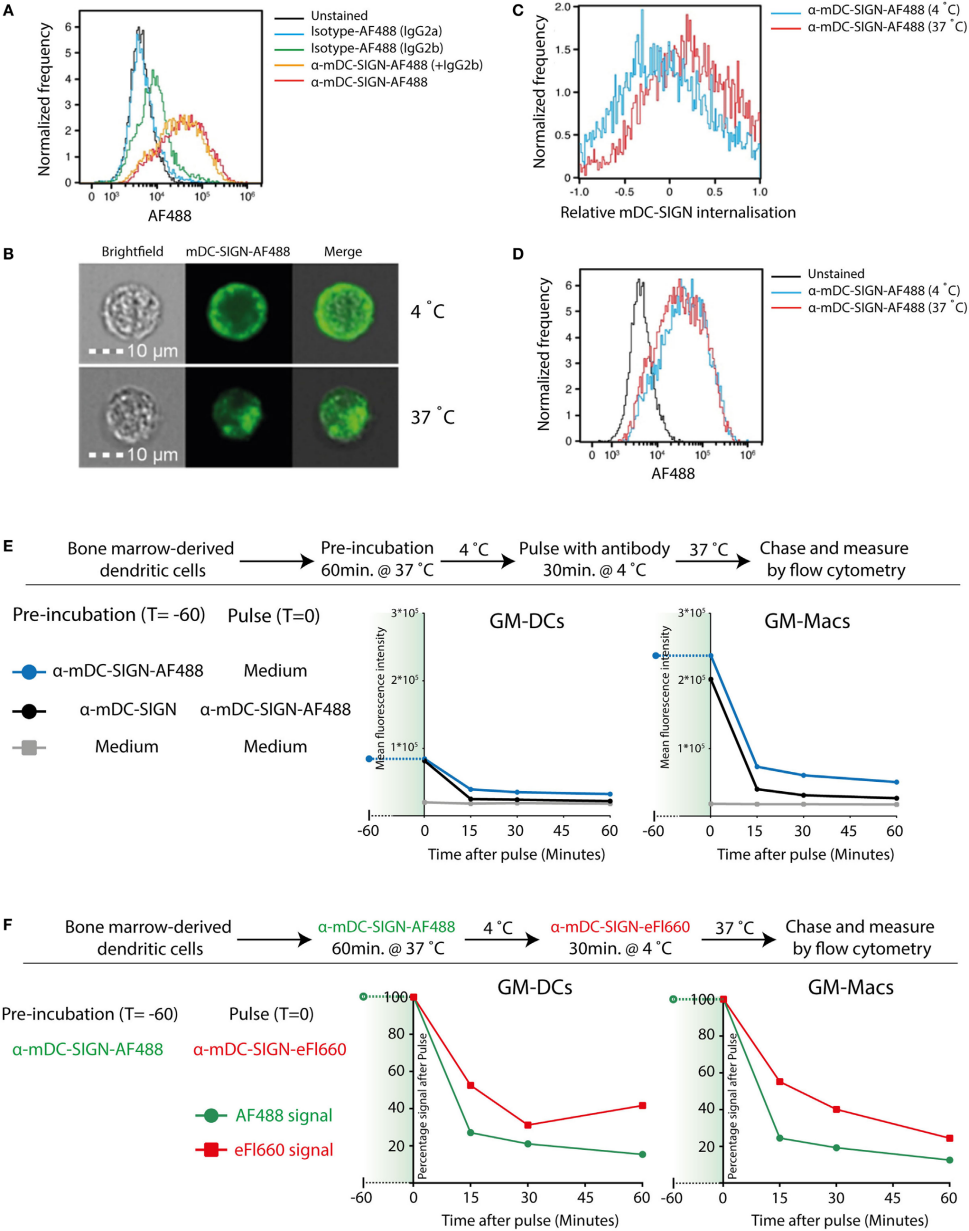
Mouse DC-SIGN (mDC-SIGN) on GM-DCs and GM-Macs is a quickly internalizing receptor for antigen processing. **(A)** Anti-mDC-SIGN binding to bone marrow-derived dendritic cells (BMDCs) cannot be blocked by isotype-IgG pre-incubation. **(B)** Example of ISX Image Stream data of mDC-SIGN-AF488 binding and internalization on BMDCs. **(C)** Upon binding, mDC-SIGN is quickly internalized at 37°C for 1 h. **(D)** Anti-mDC-SIGN-AF488 fluorescence does not increase after 1 h at 37°C, suggesting either a balance in uptake and degradation or an absence of continued uptake. Experiments representative of two individual experiments. **(E)** Pulse-chase experiments show that pre-incubation of BMDCs with unlabeled anti-mDC-SIGN (clone MMD2) for 1 h at 37°C does not abrogate pulse binding of labeled anti-mDC-SIGN-AF488 (clone MMD3). In addition, fluorescent signal is quickly reduced as the antibody is internalized and degraded in both GM-DCs and GM-Macs. **(F)** BMDC labeling 1 h with anti-mDC-SIGN-AF488 (clone MMD3) before pulse staining with anti-mDC-SIGN-eFluor660 (clone MMD3) shows similar availability of membrane-bound mDC-SIGN molecules after internalization by the first AF488-labeled antibody.

To investigate whether mDC-SIGN behaves as an internalizing receptor like hDC-SIGN, we determined the endocytic capacity of BMDCs by imaging flow cytometry. Given the sequence similarity of the different family members in the DC-SIGN family, we selected a set of monoclonal antibodies that have been previously demonstrated to specifically recognize either mDC-SIGN (MMD2/MMD3) or hDC-SIGN (AZN-D1) (4, 20, 27). Fluorescently labeled mDC-SIGN antibody (α-mDC-SIGN-AF488) recognizes mDC-SIGN, which is Fab dependent as it cannot be blocked by pre-incubation with IgGs or FC-block for 30 min (Figure 2A), as previously described (20). Using imaging flow cytometry, we can discriminate between membrane-bound fluorescence and intracellular fluorescence and thereby follow internalization of the receptor (Figure 2B). To analyze the capacity of mDC-SIGN antibody to internalize *in vitro*, we incubated GM-CSF cultured BMDCs with anti-mDC-SIGN antibody at either 4 or 37°C for an hour. We observed clear internalization of the fluorescently labeled mDC-SIGN antibody after 1 h incubation at 37°C compared with 4°C, when cells are metabolically inactive (Figure 2C; red line and blue line, respectively). Interestingly, no obvious accumulation of fluorescent signal is observed when the fluorescently labeled antibody is continually present for an hour (Figure 2D). This suggests that mDC-SIGN is either not recycled within this time window to accumulate more antibody intracellularly or the degradation of antibody is balanced by continuous uptake. To examine this, we tested whether mDC-SIGN was available on the membrane for antibody binding after 1 h of internalization with mDC-SIGN targeting antibody. Pulse-chase experiments revealed quick degradation of the fluorochrome after pulse (Figure 2E; blue line), which could not be blocked by pre-incubation with the same antibody (black line). When mDC-SIGN-AF488 fluorescently labeled antibody was incubated for 1 h and allowed to internalize at 37°C, unbound membrane-bound mDC-SIGN molecules were still available for staining with a second mDC-SIGN-eFluor660 antibody (Figure 2F, green lines and red lines, respectively). Also, the secondary antibody was internalized and degraded in a similar fashion to the mDC-SIGN-AF488 antibody. The fact that mDC-SIGN was apparently available for antibody binding after 1 h of internalization shows that mDC-SIGN is either recycled or newly synthesized. Taken together, these data suggest that mDC-SIGN has endocytic capacity as previously described for hDC-SIGN (29), but quickly resurfaces on the cell membrane for binding and uptake.

**Figure 3:**
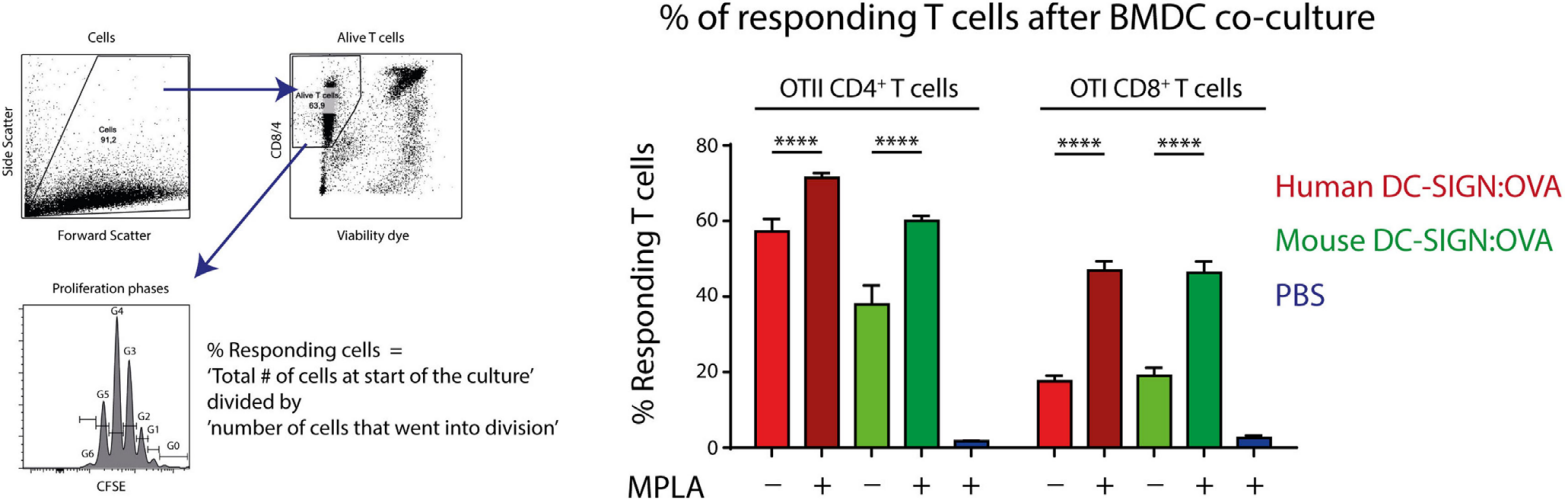
Antigen targeting to mouse- and human DC-SIGN (hDC-SIGN) on bone marrow-derived dendritic cells (BMDCs) leads to internalization, processing, and presentation to antigen-specific CD8^+^ and CD4^+^ T cells. GM-CSF cultured hDC-SIGN BMDCs pulsed with 1 μg/ml α-mDC-SIGN:OVA or α-hDC-SIGN:OVA (1 h 37°C, with Fc block) presents processed antigen to CFSE-labeled OTI CD8^+^ or OTII CD4^+^ T cells after 3 days of co-culture. Also, the TLR4 ligand, monophosphoryl lipid A (MPLA) consistently boosts antigen presentation. T cell proliferation is represented as percentage responding cells (“calculated cells at the start of culture”/“number of cells that went into division” × 100). Data represented as mean ± SEM, co-cultured in triplicates (analysis of variance with Tukey *post hoc* ****P < 0.0001), representative of three individual experiments.

Since hDC-SIGN is able to route internalized antigen to MHC class I and II complexes for presentation to T cells (5), we investigated the antigen-presenting capacity of mDC-SIGN^+^ DCs *in vitro*. GM-CSF cultured hDC-SIGN transgenic BMDCs express both mDC-SIGN and hDC-SIGN (Figure 1), allowing the comparison between these receptors using the same bone marrow culture. hDC-SIGN BMDCs pulsed with mDC-SIGN:OVA or hDC-SIGN:OVA targeting antibody were able to internalize, process, and present antigen to OTI CD8^+^ or OTII CD4^+^ T cells (Figure 3). In addition, the TLR4 agonist and known inducer of cross-presentation to CD8^+^ T cells (30), monophosphoryl lipid A (MPLA), significantly boosted antigen presentation to CD4^+^ and CD8^+^ T cells, with a more pronounced effect on CD8^+^ T cells (Figure 3). The antigen presentation capacity of mDC-SIGN using wild-type BMDCs resulted in comparable results, whereas T cell activation by hDC-SIGN:OVA was abolished in WT BMDCs lacking hDC-SIGN (Figure S1 in Supplementary Material). Hence, both mDC-SIGN and hDC-SIGN are capable of antigen presentation to CD4^+^ T cells and cross-presentation to CD8^+^ T cells *in vitro*, which can be boosted by TLR4 activation through MPLA. These data clearly support mDC-SIGN as an endocytic receptor for antigen presentation to T cells, similar to hDC-SIGN.

**Figure 4:**
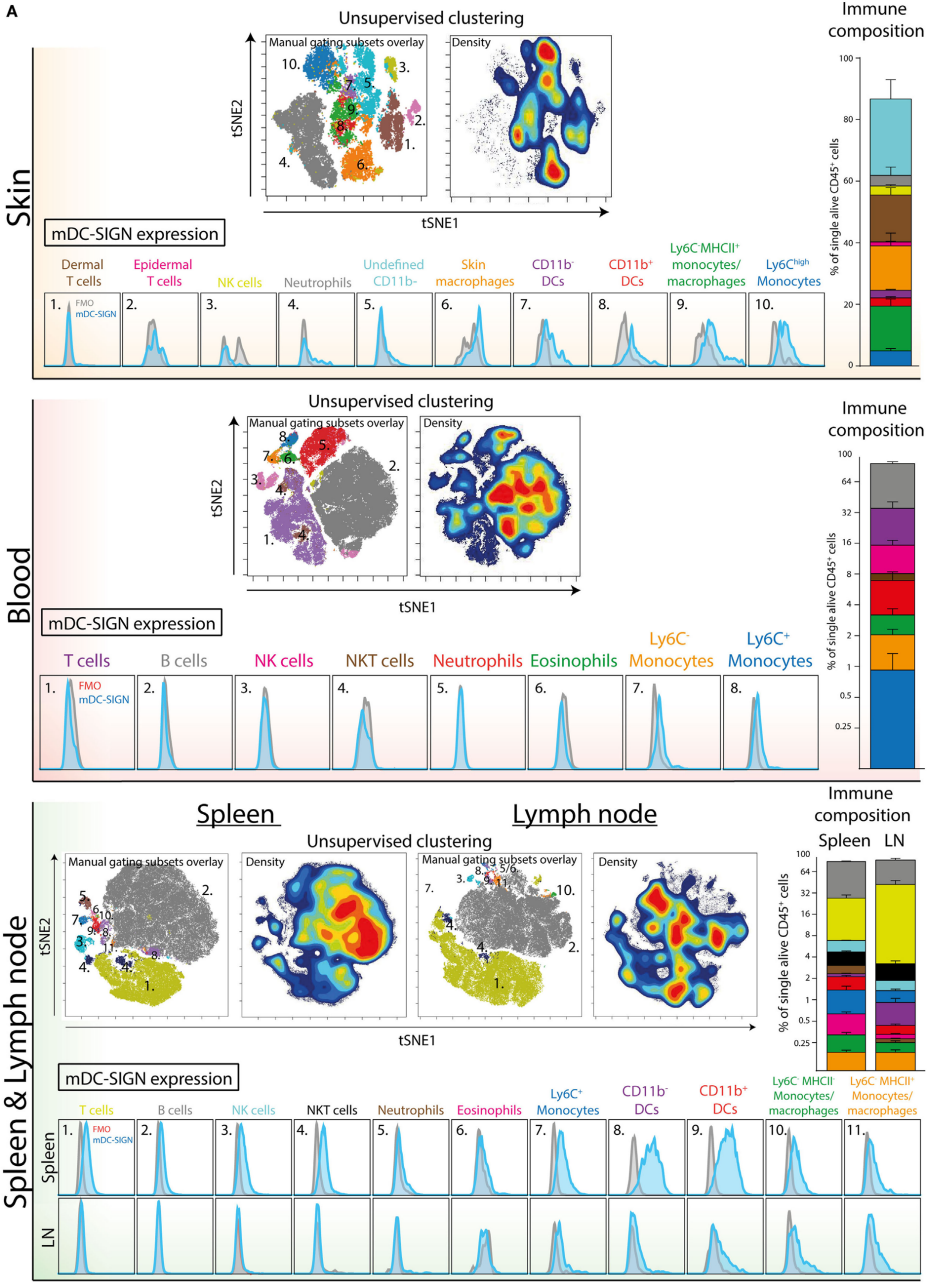

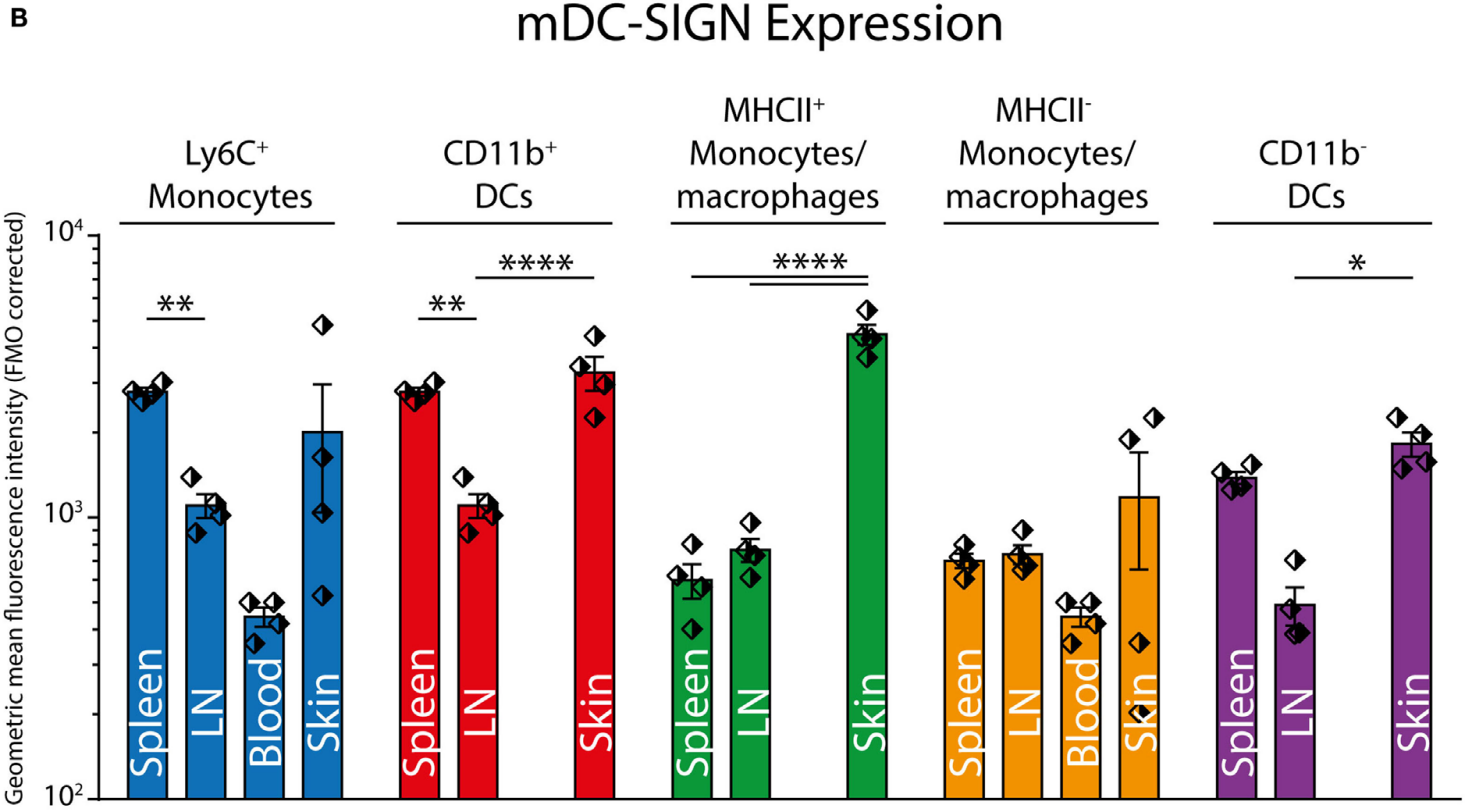
Unsupervised clustering (tSNE) of total CD45^+^ cells distinguishes organ-specific immune cell subsets expressing mDC-SIGN. **(A)** Unsupervised clustering (tSNE) of alive single CD45^+^ cells based on high-dimensional flow cytometry into two dimensions allows the distinction of immune subsets and reconstruction of cell frequency (density plot) within the immune composition. Expression of mDC-SIGN (blue histogram) is generally expressed by monocytes/macrophages in the skin and spleen. Dendritic cells in the spleen express clear levels of mDC-SIGN, but expression is less pronounced in the skin-draining lymph nodes (LNs). **(B)** mDC-SIGN is highly expressed on myeloid cells and in particular monocytes/macrophages and DCs in the skin. Also, expression is high on CD11b^−^ and CD11b^+^ DCs in the spleen, compared to the LN. Data in the bar graphs represented as mean ± SEM (*N* = 4, representative of two individual experiments).

Next, we aimed to explore mDC-SIGN expression on the major immune subsets in the skin, spleen, LN, and blood of C57/Bl6 mice. Using a 12-color flow cytometry panel including directly labeled antibodies against CD45, CD3, B220, NK1.1, Ly6C, GR-1, CD11b, CD11c, MHCII, CCR2, mDC-SIGN, and a viability dye, we could dissect the major immune population present in blood, spleen, skin, and skin-draining LN (i.e., lateral inguinal LN). To distinguish the populations, we applied tSNE unsupervised clustering as previously described (31), using the online analysis platform Cytobank. The output in Figure 4 represents all alive CD45^+^ cells with high-dimensional data in a two-dimensional plot (tSNE1 vs. tSNE2). Cells that are similar in marker expression are clustered together in space. This approach prevented us from overlooking subpopulations of immune cells in the tissue, while developing proper manual gating strategies (Figure S2 in Supplementary Material). In addition, by manually gating we could assimilate individual subsets into a clear immune composition of the tissue (Figure 4). The immune composition of the skin seems to be largely dominated by skin macrophages/monocytes [CD11b^+^Ly6C^−^GR-1^−^ (orange)], dermal T cells [NK1.1^−^ CD3^int^ (brown)], and CD11c^−^ APCs [CD11b^+^Ly6C^−^GR-1^−^CD11c^−^MHCII^+^ (green)]. Interestingly, the CD11b^+^ DCs and the CD11c^−^ APCs could only be distinguished by CD11c expression and could not be further subdivided based on the markers used in this panel. Hence, they are not distinctly separated in the tSNE analysis, suggesting that these cell types are similar based on the markers used. The immune composition of LNs, spleen, and blood is mainly dominated by lymphoid cells like B-, T-, and NK cells (gray, purple, and pink, respectively). Subsequent gating showed low but clear mDC-SIGN expression mostly on Ly6C^high^ monocytes (CD11b^+^Ly6C^high^GR-1^int^), Ly6C^−^ monocytes/macrophages (CD11b^+^Ly6C^−^), and CD11b^+^ and CD11b^−^ DCs (MHCII^+^CD11c^+^) in the skin and spleen (Figure 4A). Additional gating strategies to identify splenic CD11b^−^ and CD11b^+^ DCs (Figure 4; in purple and red) resulted in similar expression levels (Figure S3 in Supplementary Material). Lymphoid cell types and granulocytes like neutrophils and eosinophils were generally devoid of mDC-SIGN, although T-, NK-, and NKT cells showed very low levels of mDC-SIGN staining in the spleen. Expression on DCs was clearly present in the spleen, but significantly lower in the LN, while mDC-SIGN was absent on immune cells in blood or low on blood monocytes (Figure 4B).

**Figure 5:**
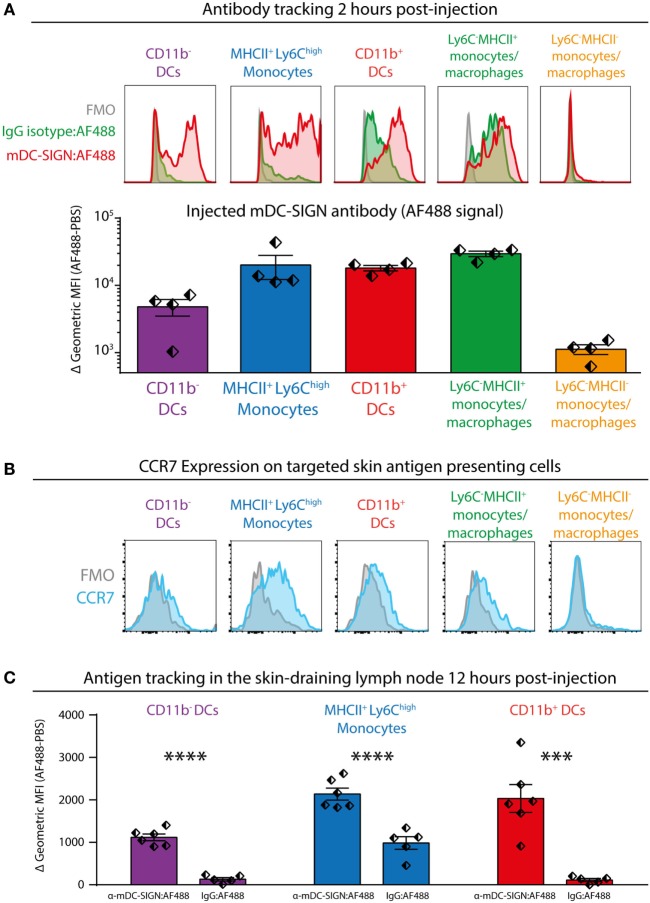
Subcutaneous injection of anti-mDC-SIGN antibody targets skin mDC-SIGN^+^ antigen-presenting cells (APCs) primarily in the skin. **(A)** 2 h after subcutaneous injection of fluorescently labeled anti-mDC-SIGN in adjuvant (MF59/AddaVax with agonistic anti-CD40 antibody) shows targeting of skin APCs. mDC-SIGN antibody shows particular targeting of MHCII^+^Ly6C^high^ monocytes, CD11b^−^ and CD11b^+^ dendritic cells. Red/green = fluorescent signal, gray = fluorescence-minus-one (FMO) negative control. **(B)** Expression of CCR7 on *ex vivo* isolated skin-derived antigen-presenting cells (APCs) 2 h after subcutaneous injection of anti-mDC-SIGN antibody. **(C)** Anti-mDC-SIGN-AF488 antibody-labeled APCs can be found in the skin-draining lymph node (LN) 12 h after injection. Data represented as mean ± SEM, analysis of variance with Tukey *post hoc* ***P < 0.001, ****P < 0.0001, representative of two individual experiments.

To investigate the potential of mDC-SIGN^+^ APCs to facilitate adaptive immunity, we injected fluorescently labeled mDC-SIGN targeting antibody and isotype control antibody subcutaneously in the skin combined with adjuvant (agonistic CD40 in MF59/AddaVax emulsion) (32). Using similar gating strategies as previously shown, injected mDC-SIGN targeting antibody can be found on mDC-SIGN^+^ APCs in the skin within 2 h after injection (Figure 5A). Notably, fluorescent IgG2c isotype control antibody was mostly bound by CD11b^+^Ly6C^−^CD11c^−^MHCII^+^ monocytes/macrophages and to some extend by CD11b^+^ DCs, but not CD11b^−^ DCs and moDCs (Figure 5A). Of note is the high level of signal from targeted APCs *in situ* compared with *ex vivo* stained skin APCs, which likely reflects the rapid turnover rate of the mDC-SIGN molecule, leading to signal accumulation in the 2 h after injection of the antibody. To distinguish between LN-homing DCs and APCs unable to migrate to LNs, we stained CCR7 on isolated APCs, 2 h after subcutaneous antibody injection. We could verify CCR7 expression on all DC subsets, including CD11b^+^Ly6C^+^MHCII^+^ moDCs, although expression levels were low (Figure 5B). To analyze the fate of the mDC-SIGN targeting antibody in the draining LNs, we isolated skin-draining LNs 12 h after subcutaneous injection of fluorescently labeled mDC-SIGN targeting antibody and analyzed the content of targeted DCs. CD11b^−^ DCs, moDCs, and CD11b^+^ DCs contained significantly more skin-injected mDC-SIGN targeting antibody compared with IgG control antibody (Figure 5C). Direct targeting of injected antibody to the skin-draining LN did not occur within 2 h after injection (Figure S4 in Supplementary Material), suggesting that these cells are derived from the periphery and are not labeled through direct drainage to the LN. Importantly, labeling was the most proficient in CD11b^+^ DCs and moDCs (Figure 5C).

**Figure 6:**
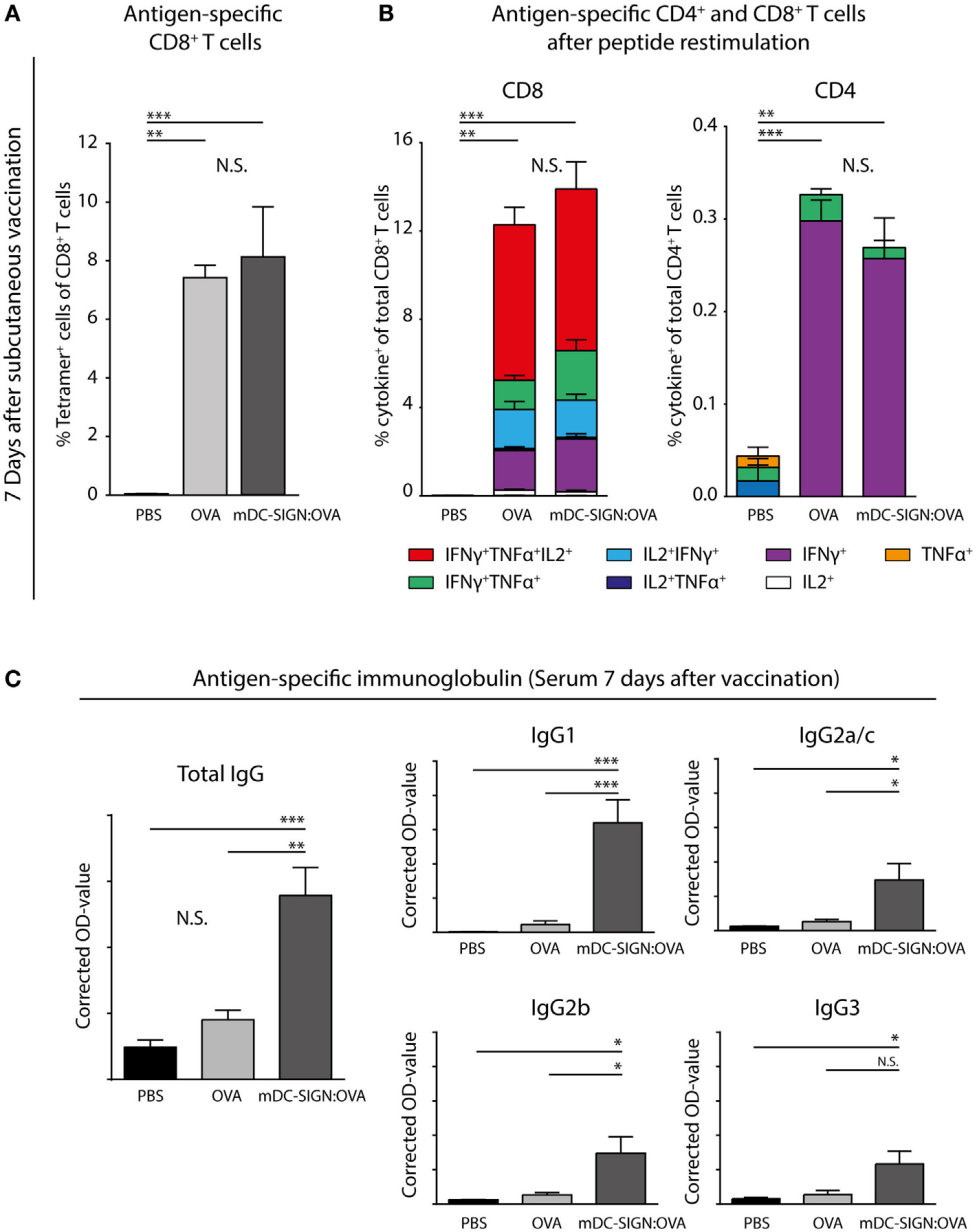
One subcutaneous dose of mDC-SIGN-OVA in adjuvant leads to antigen-specific CD8^+^ and CD4^+^ T cell responses and an enhanced isotype-switched OVA-specific antibody response. **(A)** Antigen-specific CD8^+^ T cells in splenocytes 7 days after vaccination as measured by H2-kb-SIINFEKL tetramer staining. **(B)** T cell re-stimulation through incubation with cognate antigen and intracellular cytokine staining reveals polyfunctional antigen-specific CD8^+^ and CD4^+^ T cell responses. **(C)** Antigen-specific antibody capture ELISA reveals antigen-specific immunoglobulin production in the serum of mice vaccinated with mDC-SIGN-OVA (7 days after vaccination; 1:400 serum dilution). All data represented as mean ± SEM (*N* = 5 per group). Two-way analysis of variance with Tukey *post hoc*; *P < 0.05, **P < 0.01, ***P < 0.001, and ****P < 0.0001. Graphs are representative of two individual experiments.

To determine whether *in vivo* targeting of antigen to mDC-SIGN induces adaptive immunity, subcutaneous vaccination using mDC-SIGN targeting antibody-coupled to ovalbumin (OVA) protein, with AddaVax-containing adjuvant was assessed. After 7 days, spleens and serum from s.c. vaccinated mice were collected to measure the generation of the adaptive immune response. A single dose of adjuvanted mDC-SIGN targeting antibody resulted in the generation of *de novo* antigen-specific CD8^+^ T cells (Figure 6A) and CD4^+^ T cells (Figure 6B). Antigen (OVA)-specific CD8^+^ T cells obtained high polyfunctionality as measured by intracellular cytokine staining after peptide re-stimulation (Figures 6A,B). Indeed, up to 50% of antigen-specific CD8^+^ T cells are TNFα, IFNγ, and IL-2 triple-producers upon antigen-specific re-stimulation. Interestingly, while targeting mDC-SIGN did not show significant differences in the quantity or quality (i.e., cytokine production) of the T cell responses compared with the whole protein ovalbumin, clear differences were observed in the humoral response in mDC-SIGN-OVA vaccinated mice 7 days after vaccination. OVA-specific IgG antibody titers (IgG1, IgG2a/b/c, and IgG3) after 7 days were particularly high when the antigen was targeted to mDC-SIGN (Figure 6C). Notably, the bias for B cell responses was not dependent on Fc-mediated uptake in the skin as IgG2c-OVA contributed significantly to the induction of T cell responses, while significantly less OVA-specific antibody responses were measured (Figure S4 in Supplementary Material). As such, mDC-SIGN^+^ APC targeting in the skin induces strong humoral responses while retaining cellular responses, further potentiating vaccination potential.

## Discussion

The C-type lectin receptor DC-SIGN has been described as a crucial innate immune receptor involved in a plethora of immunological processes, including the recognition of pathogen-derived ligands and self-glycoproteins, intracellular signaling, antigen processing and presentation, and activation of T cells (1). However, research on its physiological role *in vivo* has been hampered because the lack of a true ortholog in the murine genome (16). Nonetheless, CD209a (also known as SIGNR5) has been coined as mouse DC-SIGN (mDC-SIGN) because of its overlapping expression patterns and localization in the genome. Here, we show expression of mDC-SIGN on GM-CSF cultured bone marrow-derived macrophages (GM-Macs) and dendritic cells (GM-DCs) *in vitro*. Interestingly, a detailed study on the identity and transcriptome of GM-DCs and GM-Macs has suggested their similarity with *in vivo* migratory DCs and skin-resident monocyte-derived DCs/macrophages, respectively (28). This appears to be in line with our *in vivo* data that mDC-SIGN can be found on both skin-resident DCs and skin-resident monocytes. More importantly, *in vivo* GM-CSF-dependent mDC-SIGN^+^ Mo-DCs seem to arise from specific FcγIIIR^+^MHCII^+^mDC-SIGN^+^ monocytes (19). Nonetheless, while hDC-SIGN can be found on GM-CSF cultured moDCs and skin-resident CD14^+^ macrophages, the expression of hDC-SIGN^+^ on migratory DCs remains to be demonstrated, suggesting a possible discrepancy in expression patterns between mouse and hDC-SIGN.

Expression of mDC-SIGN seems to be most pronounced on APCs in organs like the spleen and skin, where pathogens are most likely to be encountered. This may suggest an endogenous role for mDC-SIGN as pattern-recognition receptor, similar to hDC-SIGN. However, hDC-SIGN functions as an important pattern-recognition receptor *via* its carbohydrate recognition domain, allowing the binding of particular sugar structures on pathogens like HIV-1, Ebola virus, *Mycobacterium tuberculosis, Candida albicans, Schistosoma mansoni, and Helicobacter pylori* (33). By contrast, mDC-SIGN does not seem to share the exact ligand specificity as hDC-SIGN (34), but has not been carefully studied in detail. Still, fucosylated Lewis x antigen, a known hDC-SIGN ligand, mediated suppression and tolerance to transplantation through mDC-SIGN^+^ monocyte-derived macrophages (35). In addition, a recent study on mDC-SIGN functioning during schistosome egg infection revealed the capacity of mDC-SIGN to signal *via* Raf-1 depending on its carbohydrate recognition domain and affect DC functioning (36). Therefore, studies aimed at investigating DC-SIGN functioning *in vivo* that rely on recognition of specific glycan structures should take the ligand-binding specificity into consideration, as well as the capacity of mDC-SIGN to affect intracellular signaling. Interestingly, among the other mouse homologs, CD209b (or SIGNR1) has been most widely investigated and shares glycan-binding specificity, including Lewis antigens, with hDC-SIGN (1). Also, the glycan-binding properties of CD209b are vital to the immunological response to *C. albicans* (37), influenza (38), and pneumococcal polysaccharides (39). However, its expression by subcapsular macrophages in LNs and marginal zone macrophages more resembles the hDC-SIGN homolog L-SIGN (40).

Regardless of endogenous ligand specificity, we provide evidence that mDC-SIGN is an internalizing receptor capable of internalizing antigen, resulting in antigen presentation to T cells. However, where hDC-SIGN has been shown to be a slow-recycling receptor (29), mDC-SIGN shows quick recovery and membrane expression after internalization. Therefore, the mode of mDC-SIGN molecule membrane homeostasis is differently regulated compared with hDC-SIGN. A current limitation of the study is the use of antibodies to target a receptor, which can add Fc-mediated effects to the experimental outcome of the results. However, since many murine SIGNR molecules exist with overlapping ligand specificity, investigating the function of mDC-SIGN *in vivo* can currently only be done using highly specific antibodies. Nonetheless, our data support the paradigm that mDC-SIGN displays functional homology to hDC-SIGN, sharing expression *in vitro* and *in vivo*, endocytic capacity, and antigen presentation to CD8^+^ and CD4^+^ T cells. This is of particular interest, since C-type lectins like DC-SIGN have been implicated in T cell functioning in humans (41). In mice, mDC-SIGN^+^ moDCs and macrophages have been shown to control T cell-mediated responses to transplantation tolerance (35), cerebral malaria (42), murine schistosomiasis (43), LPS-induced system infection (20), and experimental colon inflammation (44). Also, mDC-SIGN targeting antibody to skin mDC-SIGN^+^ APCs and skin mDC-SIGN^+^ monocytes induced antigen-specific CD8^+^ and CD4^+^ T cell responses. It remains to be defined which subset migrates to the draining LN to contribute to T cell activation, especially since moDCs are not assumed to possess high migratory potential out of peripheral tissues (45). Nonetheless, targeting mDC-SIGN led to high antigen-specific antibody responses, suggesting a potency for mDC-SIGN^+^ APCs to induce germinal center B cell responses. Notably, while conventional DCs initiate T cell responses, monocyte-derived DCs specifically boost the T follicular helper program that is needed to induce potent germinal center responses *in vivo* (46). Human moDCs have been shown to direct follicular helper T cell differentiation and subsequent T-cell-dependent IgG production by B cells *in vitro* through DC-SIGN (47). Therefore, it is possible that through targeting antigen to mDC-SIGN the mDC-SIGN^+^ moDCs provide additional Tfh programming and subsequent B cell responses. Alternatively, targeted deletion of CD11b^+^ DCs, an mDC-SIGN^+^ DC subset in the murine skin readily targeted in the presented vaccination strategy, has shown to reduce humoral responses to vaccination (48). As such, the specific contribution of specific mDC-SIGN^+^ APC subsets remains to be defined.

It is this potency by DC-SIGN^+^ cells to control adaptive immunity that has stimulated research that targets this receptor for therapeutic purposes. Indeed, recent progress has been made in using hDC-SIGN to target tumor-associated antigens to DCs for T cell activation (13, 14, 49, 50). Indeed, in the hDC-SIGN:CD11c humanized mice, targeting OVA to hDC-SIGN on DCs elicited antigen-specific CD8^+^ T cell responses capable of eradicating OVA-expressing melanoma tumors (14). Since hDC-SIGN is expressed by all CD11c^+^ cells in this model and does not reflect the natural situation in humans, targeting antigen to mDC-SIGN provides a novel method to investigate hDC-SIGN-generated adaptive immune responses. Most notably, targeting moDCs through DC-SIGN could contribute to vaccine efficacy through the generation of antibody responses. The hDC-SIGN transgenic mouse model also does not express hDC-SIGN on cells that do not express CD11c, including CD11c^−^ APC and Ly6C^+^MHCII^+^ monocytes, which express mDC-SIGN. In conclusion, using mDC-SIGN as an hDC-SIGN homolog will allow further study of DC-SIGN-initiated adaptive immune responses in the context of a complex immune system *in vivo* and will aid preclinical DC-SIGN-targeting vaccination strategies. Notwithstanding, since the mDC-SIGN receptor internalization/resurfacing characteristics and ligand specificity is different from hDC-SIGN, there is still a need to investigate the hDC-SIGN molecule in its functional tetrameric form and under its proper genomic control *in vivo*.

## Ethics Statement

All experiments were approved by the Animal Experiments Committee of the VU University and performed in accordance with national and international guidelines and regulations.

## Author Contributions

SS provided contribution to the conception and design of the work, acquisition, analysis, and interpretation of data for the work, drafting of the work and revising it critically, and agreed to be accountable for all aspects of the work. LK, MC, and HK were involved in the acquisition and analysis and interpretation of the data. JO and JJ-G were involved in the drafting of the work, and contributions to the conception and interpretation of the work. JH was involved in the analysis and interpretation of the work, drafting and critically reading of the manuscript. YK was involved in the design of the work, drafting and intellectual content of the document, and accountable for the work to accuracy and integrity. All authors provided approval for publication of the content.

## Conflict of Interest Statement

The authors declare that the research was conducted in the absence of any commercial or financial relationships that could be construed as a potential conflict of interest.
