# Supplementary material for: Mouse DC-SIGN/CD209a as Target for Antigen Delivery and Adaptive Immunity
Source: Front Immunol. 2018 May 7;9:990. doi: 10.3389/fimmu.2018.00990 (PMC5949514; doi:10.3389/fimmu.2018.00990)

Supplementary figures

Supplementary figure 1 | Antigen targeting to mouse- and human DC-SIGN on BMDCs induces proliferation in antigen-specific CD8$^+$ and CD4$^+$ T cells. CD8 and CD4 T cell proliferation by hDC-SIGN was practically abolished in WT BMDCs.

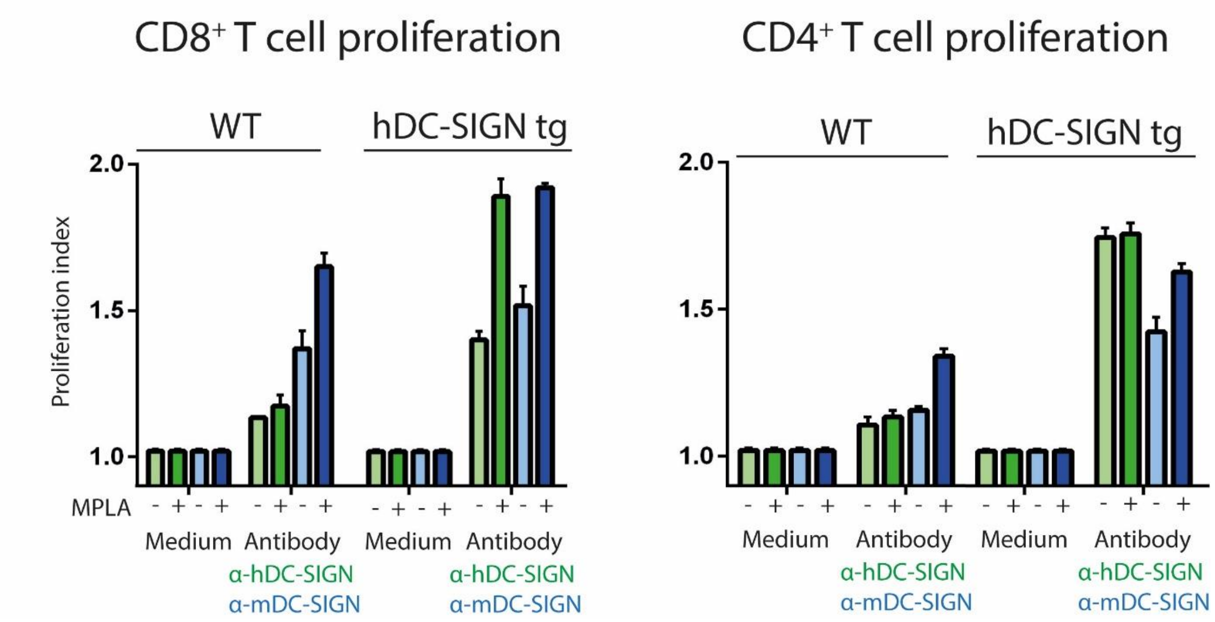

Supplementary figure 2 | Manual gating strategies for subset definition after unsupervised clustering All statistical graphs are generated from manually gated subsets from individual mouse samples as show here. Organs include blood (A), skin (B), spleen (C) and skin-draining lymph node (D). The tSNE graphs are generated using both homeostatic and vaccinated conditions as shown here.

A

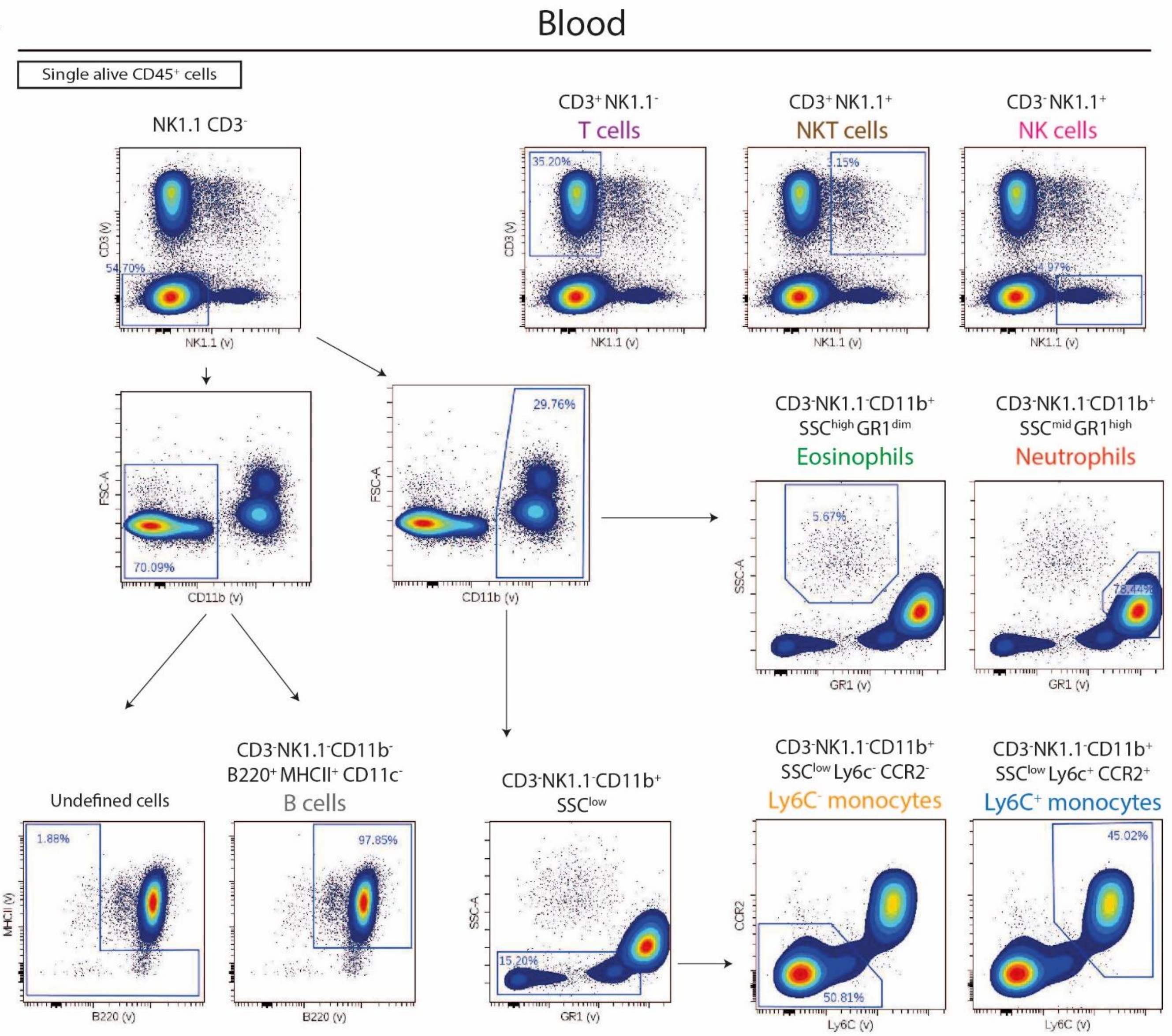

Blood
Single alive CD45+ cells
NK1.1 CD3-
CD3+ NK1.1-
T cells
CD3+ NK1.1+
NKT cells
CD3- NK1.1+
NK cells
CD3-NK1.1-CD11b+
SSChigh GR1dim
Eosinophils
CD3-NK1.1-CD11b+
SSCmid GR1high
Neutrophils
Undefined cells
CD3-NK1.1-CD11b-
B220+ MHCII+ CD11c-
B cells
CD3-NK1.1-CD11b+
SSClow
CD3-NK1.1-CD11b+
SSClow Ly6c- CCR2-
Ly6C- monocytes
CD3-NK1.1-CD11b+
SSClow Ly6c+ CCR2+
Ly6C+ monocytes
54.70%
35.20%
70.09%
29.76%
5.67%
1.88%
97.85%
15.20%
50.81%
45.02%
NK1.1 (v)
CD3 (v)
CD11b (v)
FSC-A
SSC-A
GR1 (v)
MHCII (v)
B220 (v)
CCR2
Ly6C (v)

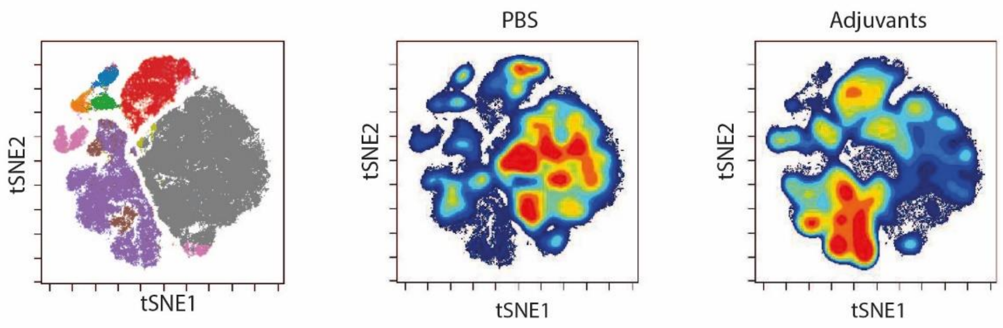

PBS
Adjuvants
tSNE1
tSNE2

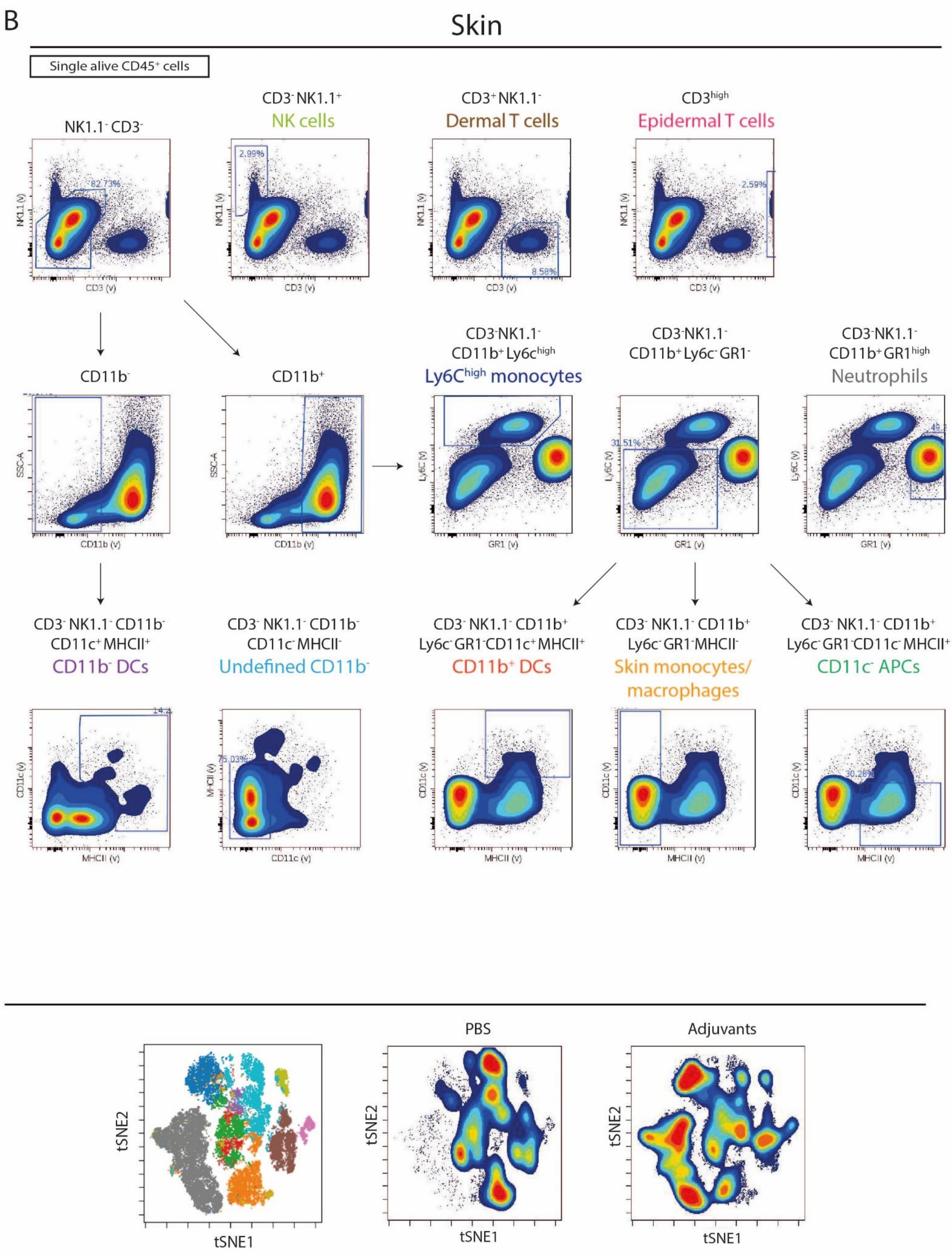
B
Skin
Single alive CD45+ cells
NK1.1- CD3-
CD3- NK1.1+
NK cells
CD3+ NK1.1-
Dermal T cells
CD3high
Epidermal T cells
82.73%
2.99%
8.58%
2.59%
NK1.1 (v)
CD3 (v)
CD11b-
CD11b+
CD3-NK1.1- CD11b+ Ly6chigh
Ly6Chigh monocytes
CD3-NK1.1- CD11b+ Ly6c- GR1-
CD3-NK1.1- CD11b+ GR1high
Neutrophils
31.51%
SSC-A
CD11b (v)
Ly6C (v)
GR1 (v)
CD3- NK1.1- CD11b- CD11c+ MHCII+
CD11b- DCs
CD3- NK1.1- CD11b- CD11c- MHCII-
Undefined CD11b-
CD3- NK1.1- CD11b+ Ly6c- GR1- CD11c+ MHCII+
CD11b+ DCs
CD3- NK1.1- CD11b+ Ly6c- GR1- MHCII-
Skin monocytes/ macrophages
CD3- NK1.1- CD11b+ Ly6c- GR1- CD11c- MHCII+
CD11c- APCs
75.03%
30.28%
CD11c (v)
MHCII (v)
PBS
Adjuvants
tSNE1
tSNE2

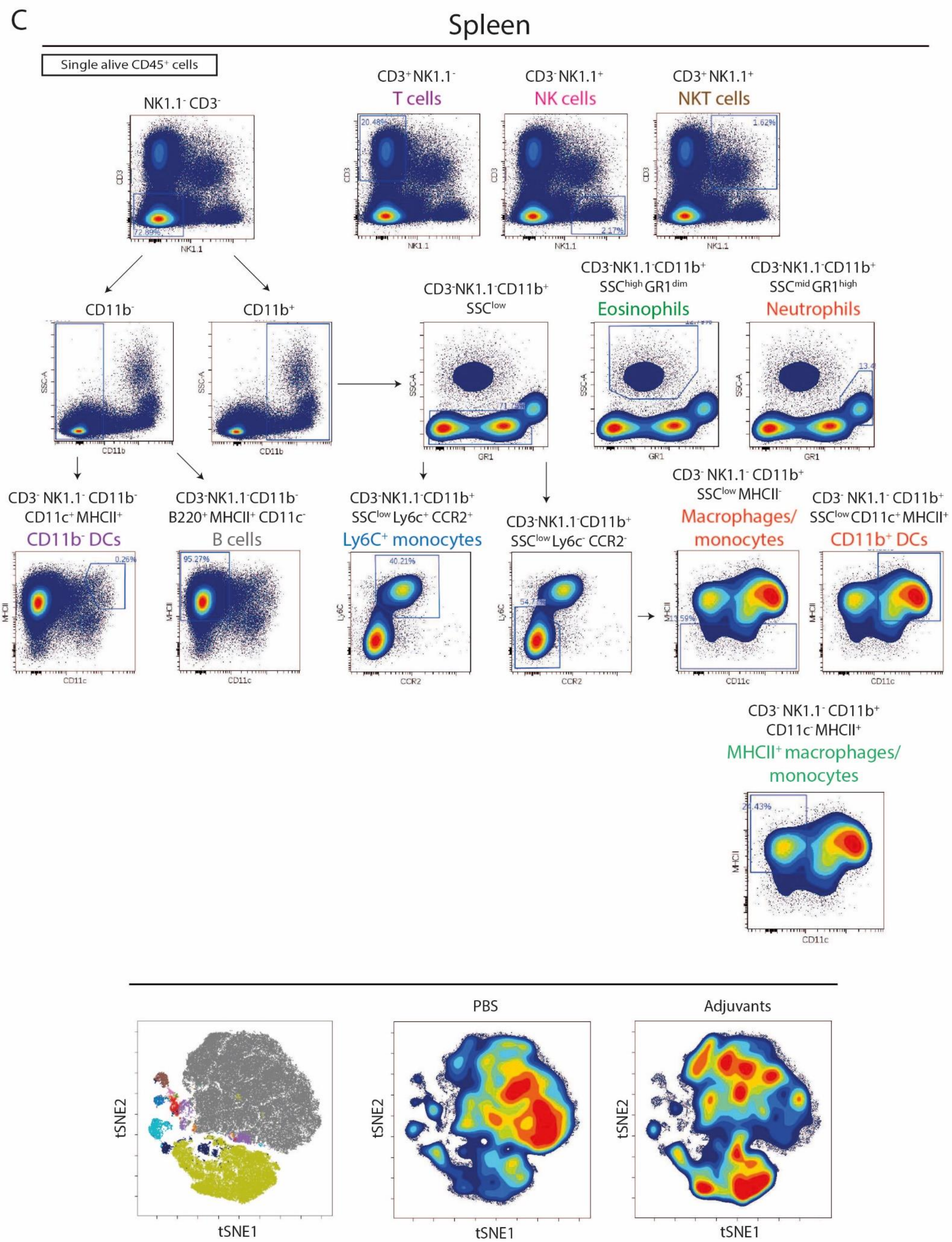
C
Spleen
Single alive CD45+ cells
NK1.1- CD3-
CD3+ NK1.1-
T cells
CD3- NK1.1+
NK cells
CD3+ NK1.1+
NKT cells
CD11b-
CD11b+
CD3-NK1.1-CD11b+
SSClow
CD3-NK1.1-CD11b+
SSChigh GR1dim
Eosinophils
CD3-NK1.1-CD11b+
SSCmid GR1high
Neutrophils
CD3- NK1.1- CD11b-
CD11c+ MHCII+
CD11b- DCs
CD3-NK1.1-CD11b-
B220+ MHCII+ CD11c-
B cells
CD3-NK1.1-CD11b+
SSClow Ly6c+ CCR2+
Ly6C+ monocytes
CD3-NK1.1-CD11b+
SSClow Ly6c- CCR2-
CD3- NK1.1- CD11b+
SSClow MHCII-
Macrophages/
monocytes
CD3- NK1.1- CD11b+
SSClow CD11c+ MHCII+
CD11b+ DCs
CD3- NK1.1- CD11b+
CD11c- MHCII+
MHCII+ macrophages/
monocytes
PBS
Adjuvants
tSNE1
tSNE2

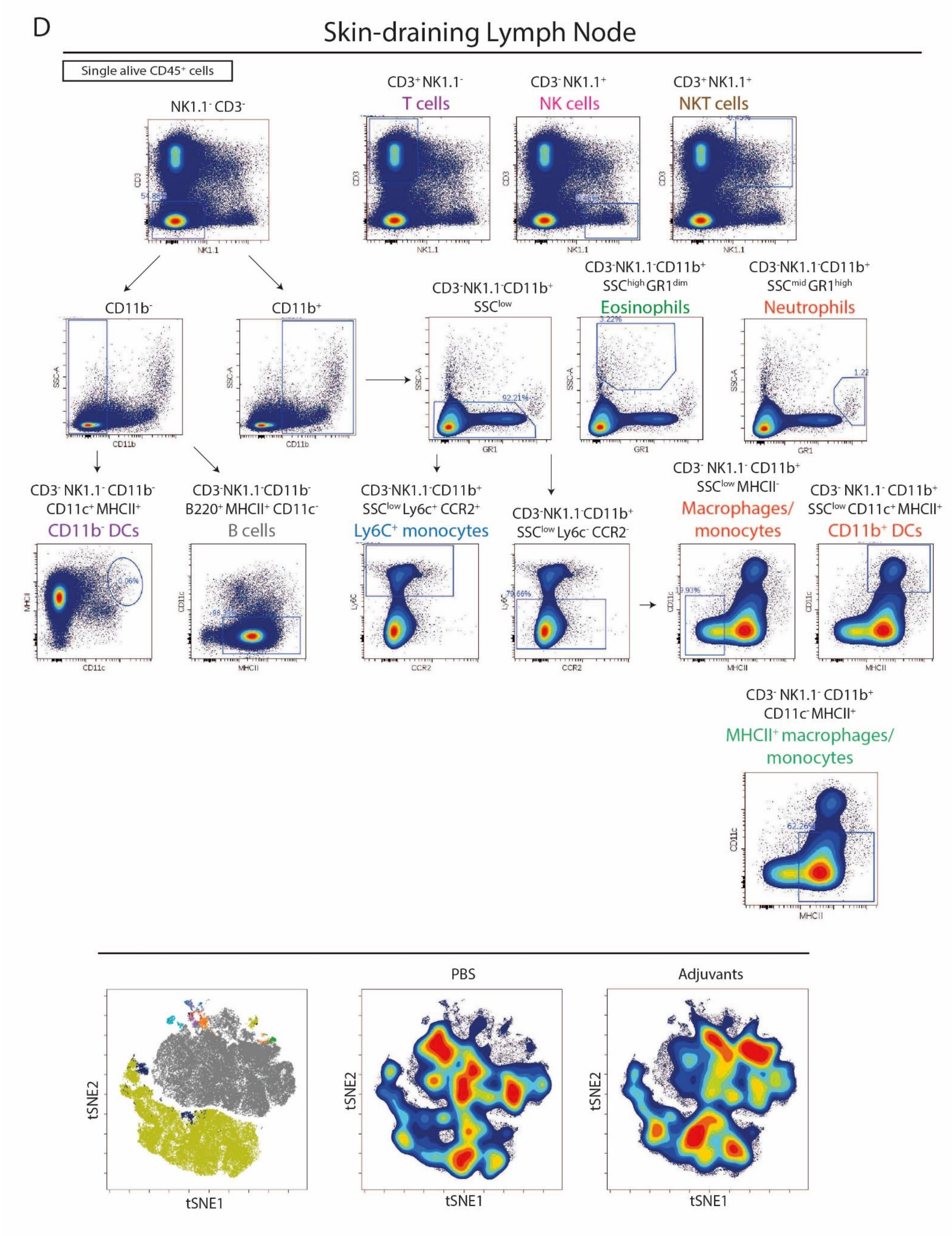
D
Skin-draining Lymph Node
Single alive CD45+ cells
NK1.1- CD3-
CD3+ NK1.1-
T cells
CD3- NK1.1+
NK cells
CD3+ NK1.1+
NKT cells
CD11b-
CD11b+
CD3-NK1.1-CD11b+ SSClow
CD3-NK1.1-CD11b+ SSChigh GR1dim
Eosinophils
CD3-NK1.1-CD11b+ SSCmid GR1high
Neutrophils
CD3- NK1.1- CD11b- CD11c+ MHCII+
CD11b- DCs
CD3-NK1.1-CD11b- B220+ MHCII+ CD11c-
B cells
CD3-NK1.1-CD11b+ SSClow Ly6c+ CCR2+
Ly6C+ monocytes
CD3-NK1.1-CD11b+ SSClow Ly6c- CCR2-
CD3- NK1.1- CD11b+ SSClow MHCII-
Macrophages/ monocytes
CD3- NK1.1- CD11b+ SSClow CD11c+ MHCII+
CD11b+ DCs
CD3- NK1.1- CD11b+ CD11c- MHCII+
MHCII+ macrophages/ monocytes
PBS
Adjuvants
tSNE1
tSNE2

Supplementary Figure 3 | Alternative gating strategies for splenic DCs shows mDC-SIGN expression on CD11b$^+$ and CD11b$^-$ splenic DCs. Classical DC gating strategies by first gating on CD3$^-$ NK1.1$^-$, then CD11c$^+$ MHCII$^+$ cells, shows similar expression of mDC-SIGN on CD11b$^-$ and CD11b$^+$ splenic DCs.

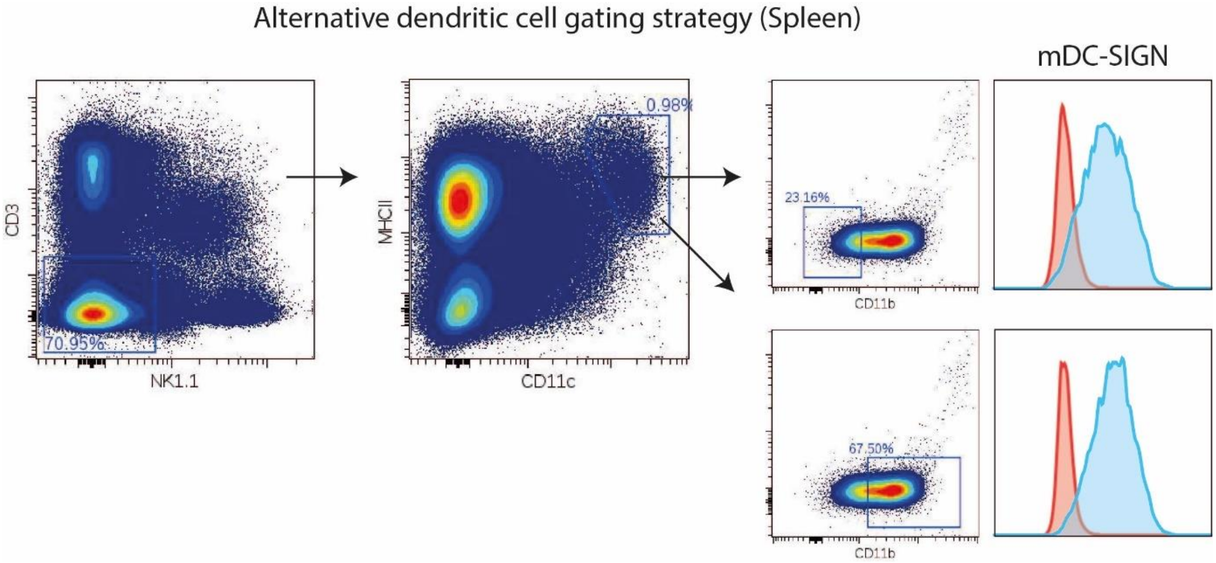

Supplementary figure 4 | Subcutaneous injection of mDC-SIGN antibody primarily targets skin-resident APCs After 2 hours of subcutaneous vaccination with fluorescently labeled anti-mDC-SIGN in adjuvant (MF59/AddaVax with agonistic anti-CD40 antibody) shows targeting of skin APCs. Upper row represents mDC-SIGN expression 2 hours after adjuvant injection without labeled antibody, while the lower row shows fluorescent signal 2 hours after adjuvant injection with labeled antibody. Blue = fluorescent signal, Red = FMO negative control

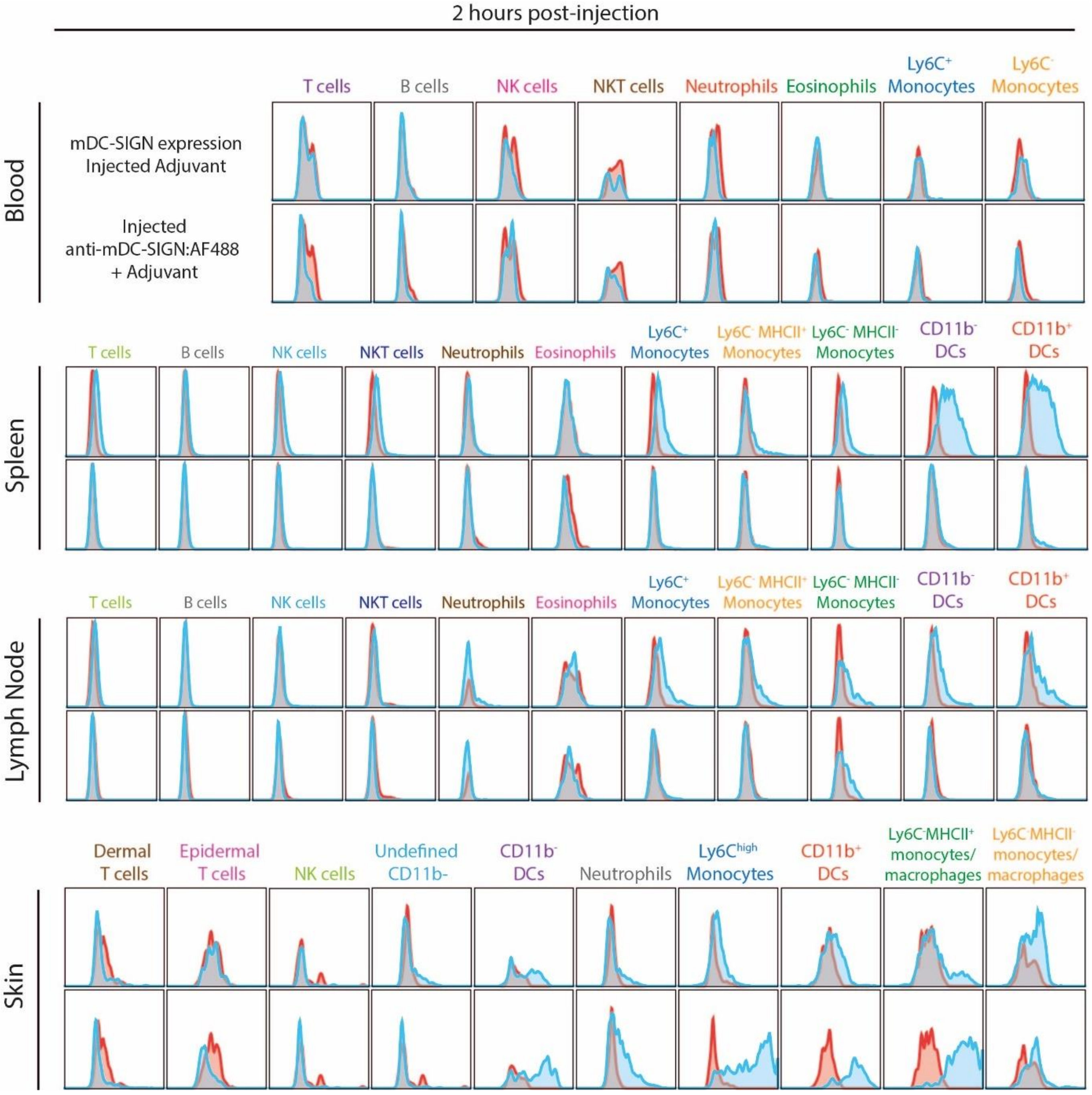

Supplementary Figure 5 | Vaccination with IgG2c and mDC-SIGN antibodies shows mDC-SIGN-specific bias towards humoral responses compared to IgG2c control antibody. Seven days after subcutaneous injection of 25ug anti-mDC-SIGN:OVA antibody or IgG2c:OVA isotype control antibody (+agonistic CD40 in 1:1 MF59/AddaVax) shows induction of antigen-specific $CD8^+$ T cell responses and humoral responses. Targeting mDC-SIGN significantly generates more antigen-specific antibodies, while the isotype control generates more antigen-specific $CD8^+$ T cells.

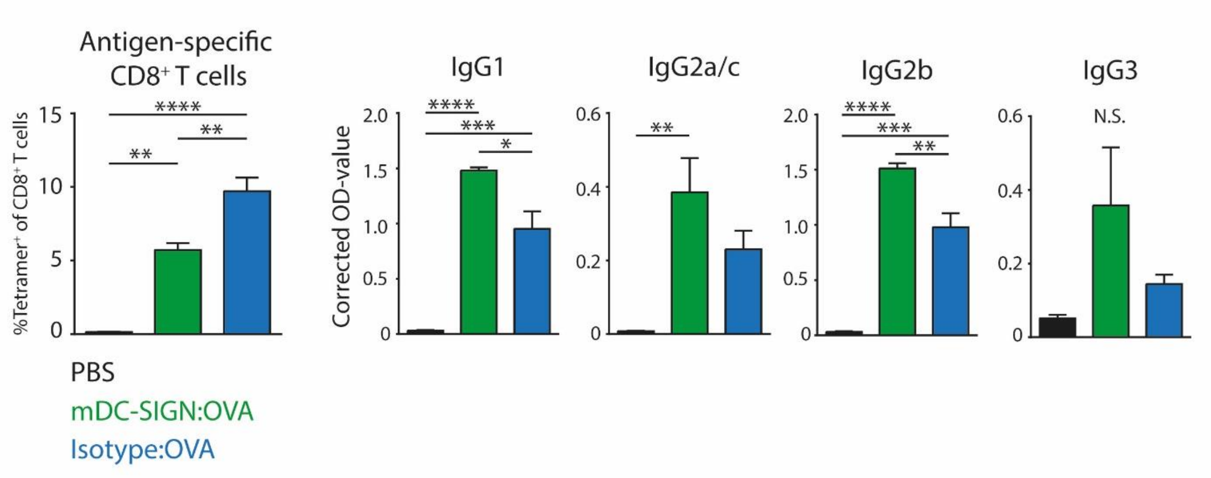

Supplement: Supplementary file 1 [file Data_Sheet_1.PDF]
